# Corrigendum: A generalized quantitative antibody homeostasis model: antigen saturation, natural antibodies and a quantitative antibody network

**DOI:** 10.1038/cti.2017.19

**Published:** 2017-05-19

**Authors:** József Prechl

**Correction to:** *Clinical & Translational Immunology* (2017) **6**, e131; doi:10.1038/cti.2016.90; published online 24 February 2017

All instances in the manuscript where the text B1I (cells) appears should be read as BI.

The publication date of the PDF has been amended to 24 February 2017.

